# “Feed-and-wrap” technique versus deep sedation for neonatal magnetic resonance imaging: a retrospective comparative study

**DOI:** 10.1007/s00330-024-10777-6

**Published:** 2024-05-07

**Authors:** André Lollert, Kim Sophie Frey, Christian Hoffmann, Markus Herbst, Jochem König, Isabella Schmeh, Frank Dette, Gundula Staatz

**Affiliations:** 1 Department of Diagnostic and Interventional Radiology, Section of Pediatric Radiology, Medical Center of the Johannes Gutenberg-University, Mainz, Germany; 2 Institute of Medical Biostatistics, Epidemiology and Informatics, Medical Center of the Johannes Gutenberg-University, Mainz, Germany; 3 Department of Pediatrics and Adolescent Medicine, Medical Center of the Johannes Gutenberg-University, Mainz, Germany; 4 Department of Anaesthesiology, Medical Center of the Johannes Gutenberg-University, Mainz, Germany

**Keywords:** Neonate, Infant, Magnetic resonance imaging, “Feed-and-wrap”, Sedation

## Abstract

**Objectives:**

Neonatal MRI is usually performed under deep sedation, which is challenging—especially in low-weight premature patients. In addition, long-term side effects, such as neurotoxicity, are of concern. An alternative to sedation is to induce natural sleep by feeding and immobilising the child, the “feed-and-wrap” technique (FWT). The objective of this study was to evaluate differences in image quality between neonates examined under sedation and by using the FWT during the first four months of life.

**Materials and methods:**

We retrospectively assessed image quality (based on a 4-point semiquantitative scale) of all MRI examinations in neonates performed at our institution between July 2009 and August 2022. Differences in image quality between examinations under sedation versus FWT were evaluated.

**Results:**

We included 432 consecutive patients, 243 (56%) using sedation and 189 (44%) using the FWT. Corrected age and body weight (mean ± SD: 3.7 ± 1.1 versus 4.5 ± 1.3 kg, *p* < 0.001) were significantly lower in the FWT group. The overall success rate in the FWT group was 95%. Image quality was slightly lower when using the FWT (mean ± SD: 3.7 ± 0.43 versus 3.96 ± 0.11, *p* < 0.001). Multivariate analysis showed a higher risk of acquiring sequences with diagnostic limitations in the FWT group (*p* < 0.001), increasing with corrected age (*p* = 0.048).

**Conclusion:**

The FWT is a highly successful method to perform MRI scans in term and preterm neonates. Overall image quality is only slightly lower than under sedation. Especially in immature low-weight preterm patients, the FWT is a reliable option to perform MRI studies without exposing the child to risks associated with sedation.

**Clinical relevance statement:**

The “feed-and-wrap” technique enables high-quality MRI examinations in neonates, including low-weight premature patients. Deep sedation for diagnostic MRI procedures in this age group, which has the risk of short- and long-term complications, can often be avoided.

**Key Points:**

- *Deeply sedating neonates for MR examinations comes with risks.*
- *Image quality is only slightly lower when using the "feed-and-wrap" technique.*
- *The “feed-and-wrap” technique is feasible even in low-weight premature infants.*

## Introduction

Magnetic resonance imaging (MRI) is the modality of choice for many indications in paediatric imaging due to the lack of ionising radiation and its high soft-tissue contrast. However, drawbacks of the modality include long examination times and suboptimal image quality in case of patient motion. Despite these limitations, MRI has emerged to an important method in term, and even preterm neonates, to assess mainly neurological [1], but also oncological [2], urological [2, 3] or cardio-vascular [4] disorders. The most frequent indication to perform MRI scans in neonates is perinatal asphyxia with suspected hypoxic-ischaemic encephalopathy [5, 6].

With the increasing use of neonatal MRI, there has been a rising necessity for deep sedation to reduce motion artefacts and achieve optimal image quality. Deep sedation is defined as a depressed level of consciousness, not allowing the patient to be aroused easily, but being able to respond purposefully following repeated or painful stimuli [7]. However, sedation is challenging, especially in low-weight preterm patients, due to their unique anatomy, physiology and pharmacokinetic behaviour [8]. First, the immature airway and respiration system is very volatile to exogenous stress such as mechanical ventilation, which should thus be avoided whenever possible. Problems related to sedation, such as prolonged desaturation and airway obstruction, can also occur in term infants [9]. In addition, some MR-compatible anaesthesia machines or devices have specific minimum bodyweight limits. Second, possible long-term side effects such as neurotoxicity are of concern. These side effects of exposure to all currently available anaesthetic sedative agents (both intravenous, e.g., propofol and inhalational, e.g., sevoflurane) were demonstrated in several animal studies. Although translation to human populations did not shows clear results so far [10], avoiding pharmacological sedation is highly relevant in this specific age group to prevent the child from acute periprocedural adverse events and from possible long-term complications.

Another technique for neonatal MRI is to immobilise the child after feeding. Several approaches have been proposed, usually termed as the “feed-and-sleep”, “feed-and-swaddle”, or “feed-and-wrap” technique (FWT). The first step, which all of the aforementioned methodological descriptions have in common, is to induce natural sleep by feeding the child prior to the examination [11]. After that, wrapping or swaddling the child in blankets is performed. Furthermore, sandbags or, most frequently, an MR-compatible vacuum mattress immobilise the patient. In addition, noise attenuators and pacifiers are used [12]. There are several studies in which the FWT was applied [11, 13–15]. These studies present with inconsistency of whether to use external immobilisation devices, such as vacuum mattresses in general [15, 16], only for selected patients [11], or not at all [14]. Thus, it remains unclear if success rates might depend on the technical details. Furthermore, most of the aforementioned studies lack a comparison of image quality with corresponding examinations under sedation, or the number of patients examined under sedation was low [16].

Therefore, the objectives of this study were to evaluate differences in success rates, as well as sequence quality, between MRI examinations under deep sedation versus the FWT (including a vacuum mattress for maximal immobilisation) in a large cohort of patients.

## Patients and methods

### Patients

Approval by the local Independent Ethics Committee was not necessary due to the retrospective study design. The legal guardians of all patients provided written informed consent for the clinically indicated MRI examinations. The study population consisted of all consecutive patients who underwent at least one MRI examination during the first four months of life in our tertiary care University Medical Center from July 2009 until August 2022. We collected patient-specific data, including gender, body height and weight, prematurity, chronological and corrected age at the time of the MRI examination from the medical records. We applied corrected age for preterm (i.e., < 37 weeks of gestation) patients, calculated as chronological age minus the difference between the estimated and actual birth date, for the statistical analyses.

### MRI

MRI examinations were performed using a 1.5 T scanner (Magnetom Avanto®; Siemens Healthineers) or a 3 T scanner (Magnetom Skyra® or Magnetom Vida®, Siemens Healthineers). Appropriate coils and standard protocols, depending on the indication and body region, were used as determined by the responsible radiologist. We retrospectively documented field strength, indication, examined body region, technique (deep sedation versus FWT), total number of sequences, use of contrast agent, and total examination length for further analysis. Total examination length was defined as the time from acquisition of the first localiser sequence until the end of the last performed sequence, including possible breaks due to awakening of the patient when using the FWT.

### Periprocedural management for MRI using deep sedation

Examinations in the deep sedation group (shortened as “sedation” group throughout the further manuscript for better readability) were performed using intravenous propofol or inhalational sevoflurane (applicated via laryngeal mask) as determined by the anaesthesiologist [17]. Endotracheal intubation, if necessary, was carried out at the paediatric intensive care unit before the child was transported to the MRI scanner. Children were kept fasting prior to the examination according to national anaesthesiology guidelines [18]. In our institution, dedicated paediatric radiologists radiologically supervised neonatal MRI under sedation since 2009. For a larger sample size in the sedation group, we also included cases examined between 2009 and 2015 in our analysis, as the respective MRI protocols were equivalent to those in the FWT group.

### Periprocedural management for MRI using the FWT

**Fig. 1:**
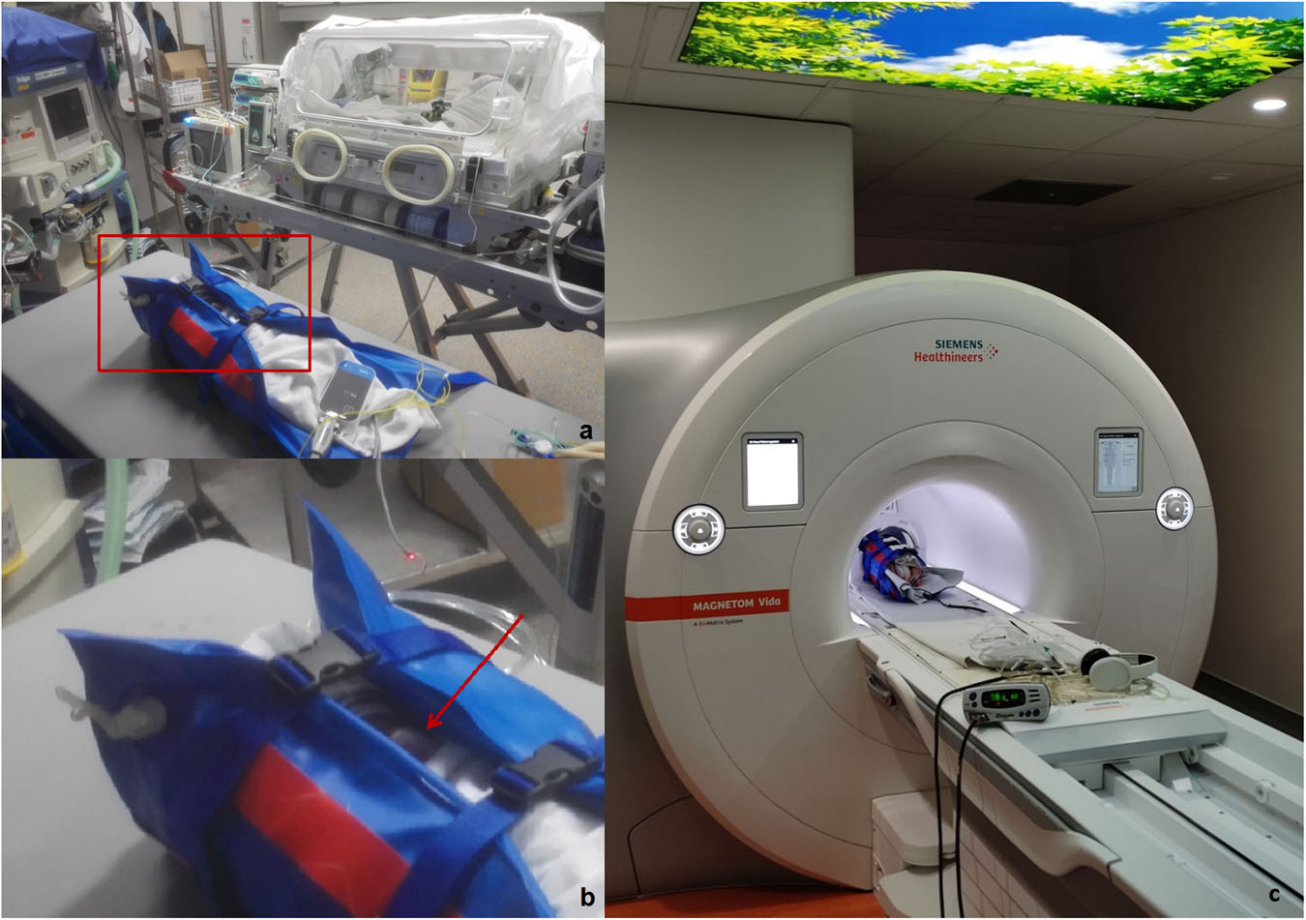
immobilisation of a premature patient in the MR preparation room. The child is transferred from the incubator into the vacuum mattress, MR-compatible monitoring is applied (**a**) and a pacifier (**b**, red arrow) is used. After vacuum has been applied, the child is taken into the MR scanner (**c**)

The FWT was introduced in 2015 in our institution. The responsible radiologist decided whether to perform the examination by using the FWT or under sedation in consensus with clinical and anaesthesiology staff. A predetermined need for contrast agent application did not exclude an FWT attempt. Patients planned for MRI using the FWT were prepared according to a standardised scheme whenever possible. A radiologist explained the whole examination setup to the parents the day before the planned time slot. We instructed the parents to keep the child awake 3 to 4 hours before the examination. Approximately 30 minutes before the examination, the children were fed, diapers were changed, all metal objects were removed, and metal-free clothes were put on. Then, we wrapped/swaddled the child into blankets with a special focus on avoiding skin-to-skin contacts (e.g., between the arms and thorax, or between the legs). We used an MR-compatible vacuum mattress (MedVac VMR433X01N, Kohlbrat & Bunz GmbH) for immobilisation in the MR unit. MR-compatible earplugs and noise attenuators (“Mini Muffs”, Natus Medical Inc) were applied for noise reduction. An MR-compatible pacifier in combination with oral glucose (5%) was offered to calm the child [19]. Vital parameters were monitored using an MR-compatible pulse oximetry system (Nonin Sensors 7500 FO, Nonin Medical Inc) throughout the examination. Figure 1 demonstrates a typical setup in the MR preparation room and scanner.

### Image and quality analysis

**Fig. 2:**
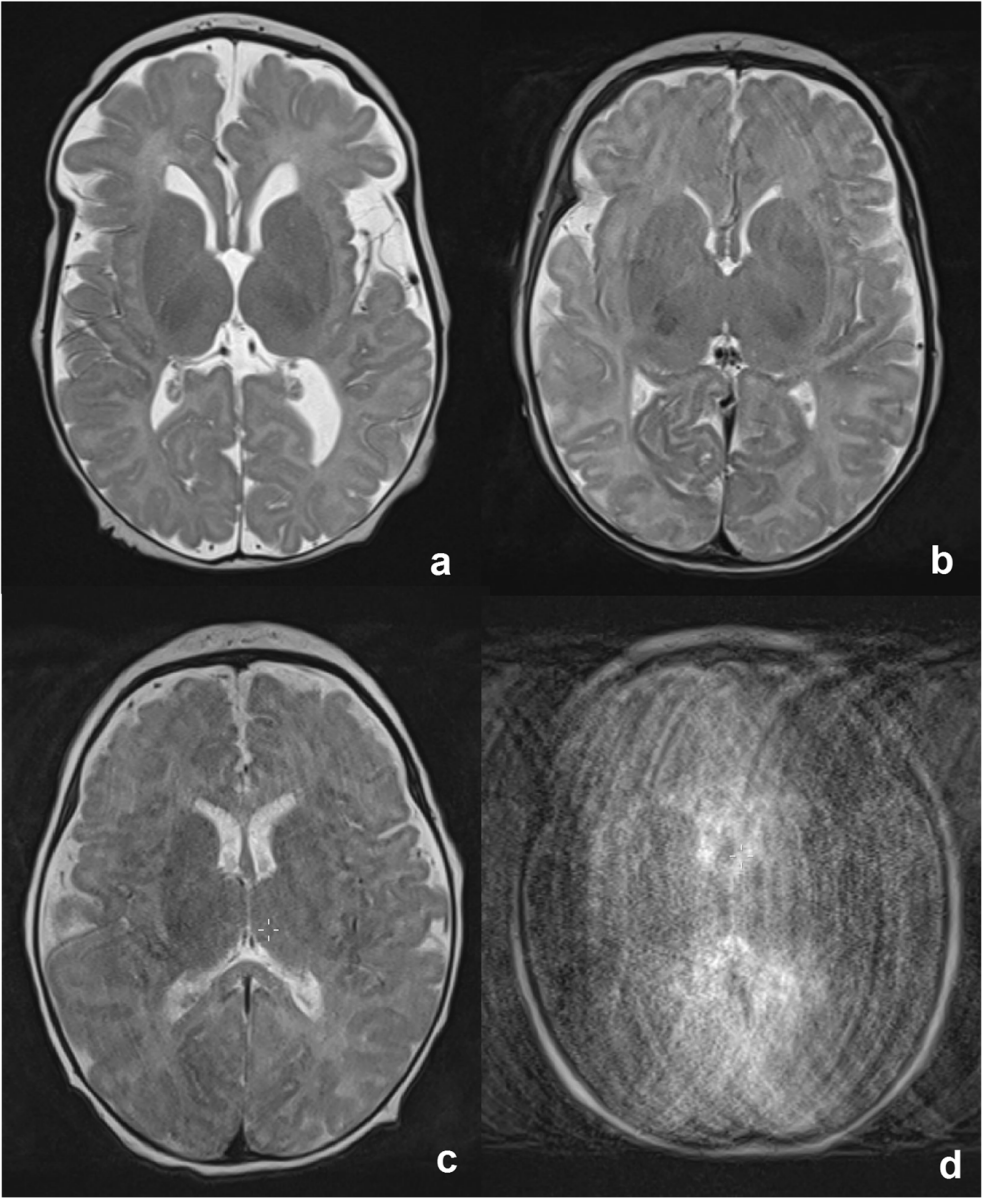
Examples of the different quality scores based on a T2-weighted turbo spin echo sequence of the brain at the level of the basal ganglia. **a** Score of 4 with excellent image quality. **b** Score of 3 with slight motion artefacts, primarily affecting the frontal lobe. **c** Score of 2 with marked artefacts. Anatomic landmarks are still visible, but details, such as cortico-medullary differentiation, are only partially assessable. **d** Nondiagnostic image with severe artefacts, corresponding to a score of 1

All sequences were retrospectively analysed by two of the authors (one paediatric radiologist with more than 10 years of experience in paediatric MRI reading, AL, and one last-year medical student, KSF) in consensus. A 4-point semi-quantitative scale was used to assess image quality, with 4 being the highest score: A score of 1 indicated severe motion artefacts, resulting in nondiagnostic images. A score of 2 indicated an image quality with marked artefacts, partially limiting diagnostics. A score of 3 was given in the case of mostly good image quality with only minor artefacts in the area of interest, predominantly not limiting diagnostics. Excellent images without artefacts or diagnostic limitations were rated with a score of 4. Figure 2 demonstrates examples of the four categories. For multivariate analyses, a sequence rated with a score of 1 or 2 was called a sequence with diagnostic limitations. Mean scores were calculated for each examination.

In addition, we assessed further factors, indicating examination quality: number of repeated sequences (including reasons for repetition), whether the main clinical question was answered, discontinuation of the examination (including reasons for termination), and if the whole examination was repeated.

### Statistics

Statistical evaluations were performed with SPSS Statistics (Statistical Package for the Social Sciences, version 27.0; IBM) or R (version 4.3, The R Foundation of Statistical Computing). A minority of patients had multiple examinations in the determined age span. As none of these examinations were performed on the same day, they were modelled as stochastically independent. Descriptive statistics were computed for all clinical and radiological parameters. Group differences were assessed using the Mann-Whitney U test, or Fisher’s exact test, as appropriate. Spearman’s rho was calculated to correlate sequence quality with corrected age. We compared both techniques with regard to the occurrence of sequences with diagnostic limitations. To this end, we fitted a negative binomial regression model (for count data) to estimate the incidence rate ratio, adjusting for age, gender and body weight as well as for the examined body region and the field strength used. The incidence rate was defined as the proportion of sequences with diagnostic limitations (i.e. sequences with a quality score of 1 or 2) per examination. A *p* value < 0.05 was considered significant in all statistical analyses, which were exploratory. Therefore, the presented *p* values were descriptive.

## Results

### Patient characteristics

**Fig. 3:**
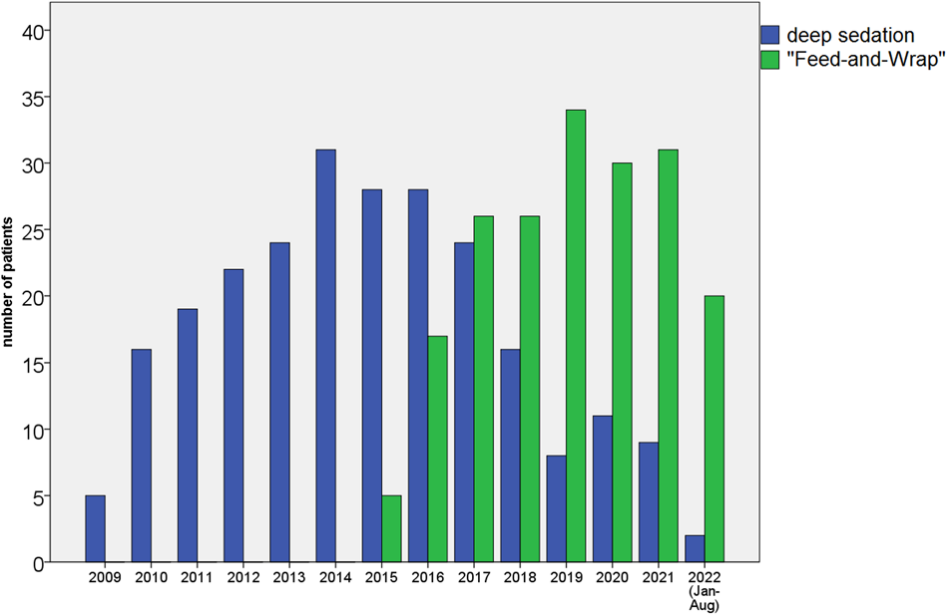
Development of examination numbers under sedation versus FWT over time. Since the introduction of the FWT in 2015, the number of examinations under sedation decreased continuously

A total of 432 consecutive patients were included, 243 (56%) using sedation and 189 (44%) using the FWT. Of these, 345 patients were examined once, 26 had two, 9 had three, and 2 patients had four examinations in the determined age span. None of the multiple examinations were performed on the same day. The main reasons for multiple examinations of a patient included oncological (i.e. re-staging) or clinical need for follow-up (e.g. follow-up of intracranial bleeding or cerebral sinus thrombosis). In 18 patients, the same technique was applied in the follow-up examination(/-s) (4 FWT/FWT, 14 sedation/sedation), in 14 patients, the technique was switched from FWT to sedation, and in 5 cases from sedation to FWT. Patient characteristics are shown in Table 1. Noticeably, patients examined by the FWT had a significantly lower age (especially when corrected for prematurity, mean 19 versus 51 days, *p* < 0.001) and weight (mean: 3.7 versus 4.5 kg, *p* < 0.001). The number of low-weight patients (≤ 3 kg) was significantly higher in the FWT group (28% versus 13%, *p* < 0.001). The lowest gestational age of a premature patient at the time of imaging was 33 + 1 weeks. This female patient successfully underwent an MRI using the FWT at a chronological age of 8 days with a body weight of 1.8 kg. Since its introduction in our institution in 2015, the number of patients examined using the FWT increased, while the number of examinations under sedation decreased (Fig. 3).

**Table 1:** Differences in patient characteristics between the anaesthesia/sedation and FWT group

	Sedation (*n* = 243)	Feed-and-wrap (*n* = 189)	Total (*n* = 432)	*p* value
**Gender**				
Female	131 (54%)	83 (44%)	214 (50%)	**0.042**
Male	112 (46%)	106 (56%)	218 (50%)	
**Age in days**				
Mean ± SD	59 ± 38	27 ± 26	45 ± 37	**< 0.001**
Corrected age* in days				
Mean ± SD	51 ± 40	19 ± 28	37 ± 39	**< 0.001**
**Prematurity**				
No	200 (82%)	156 (83%)	356 (82%)	> 0.999
Yes	43 (18%)	33 (17%)	76 (18%)	
**Body weight in kg**				
Mean ± SD	4.5 ± 1.3	3.7 ± 1.1	4.1 ± 1.3	**< 0.001**
Minimum	2.1	1.6		
Maximum	9.0	9.3		
≤ 3 kg	31 (13%)	52 (28%)	83 (19%)	**< 0.001**
> 3 kg	212 (87%)	137 (72%)	349 (81%)	
**Body height in cm**				
Mean ± SD	55 ± 5.5	52 ± 4.6	54 ± 5.3	**< 0.001**
**Ward/referring unit**				
Standard care	175 (72%)	133 (70%)	308 (71%)	0.748
Intensive care	68 (28%)	56 (30%)	124 (29%)	

Significant differences are marked in bold. *Corrected age was applied for preterm (i.e., < 37 weeks of gestation) patients and calculated as chronological age minus the difference between the estimated and actual birth date. In term patients, the corrected age is equal to the chronological age

### MRI

The main indications for MRI were neurological (e.g. perinatal asphyxia, 58%) and oncological (e.g., teratoma, neuroblastoma, 14%). Thus, brain MRI was most frequently performed (60%), followed by whole-body/multiple-body regions (18%) and abdominal examinations (13%).

MRI was completed successfully in 420/432 cases (97%). The success rates were 99% (240/243 cases) in the sedation group and 95% (180/189 cases) in the FWT group. Events that led to nondiagnostic or not completely conclusive examinations are described in detail hereafter.

In the sedation group, one examination of a 6-day-old infant had to be aborted prior to imaging due to cardiopulmonary instability. This examination was repeated 4 days later when a short protocol comprising of 3 sequences could be acquired before the child became unstable again. In another child, the examination was aborted due to dislocation of the i.v. tube with extravasation. The child consecutively awoke from sedation, and the radiologist decided to end the examination despite motion artefacts in the last sequence.

No severe periprocedural complications (i.e., cardio-respiratory problems or other potentially life-threatening events) occurred in the FWT group. However, a total of 3 examinations were aborted prior to image acquisition due to severe patient movement. In 4 cases, a few sequences could be acquired, but the radiologist suggested a repetition under sedation because the clinical question could not be answered adequately. Two of the seven aforementioned examinations were successfully repeated under sedation. Reasons for nonrepetition included refusal of consent (*n* = 1) or withdrawal of the clinical indication based on interdisciplinary consensus (*n* = 4). In two cases, the examinations were nearly complete when they had to be aborted due to patient motion. In these cases, the radiologist did not suggest a repetition because most of the clinical questions could be answered.

### Differences between the sedation and FWT group

Differences in MRI-associated data between both groups are summarised in Table 2. Total examination times were significantly shorter when using the FWT (mean 32 versus 39 min, *p* < 0.001). An additional subgroup analysis based on the use of contrast agent and body region confirmed this fact for examinations without contrast agent, as well as in head, abdominal and whole-body examinations (Supplementary Table 1). The majority of examinations consisted of brain MRI, with no significant difference between both groups (sedation: 57%, FWT: 64%). Whole-body MRI was slightly more often performed under sedation (21% versus 14%). While the total number of sequences was lower in the FWT group, the number of repeated sequences (mainly due to patient motion) was higher. Overall sequence quality was excellent when using sedation (mean quality score of 3.96), while it was still good (mean quality score of 3.7) in the majority of examinations using the FWT. Contrast agent was avoided in most of the examinations using the FWT. The number of sequences with diagnostic limitations (mean 0.07 versus 0.49), as well as the incidence rate, were lower in the sedation group. These differences were significant but low in terms of absolute numbers. A total of 2160 sequences were acquired in the sedation group, of which 2104 were rated with an optimal score of 4 (97.4%). In the FWT group, this rate was 82.6% (1265/1531 sequences). Multivariate negative binomial regression demonstrated an approximately 8-to-9-fold higher risk of acquiring a sequence with diagnostic limitations when using the FWT (Table 3). This risk was significantly higher for head examinations compared with abdominal or whole-body MRI. In addition, a significant increase in the incidence rate with increasing corrected age was shown. Furthermore, we divided the dataset into different age groups (corrected age of 50 days or lower, and beyond 50 days), because of our clinical experience that FWT MRI becomes more challenging in older infants, and due to the impression of our initial results. These data are presented in supplementary table 2. Of note, in the latter subgroup, the aforementioned clinical impression of difficulties when using the FWT in older infants had the statistical correlate of a higher incidence rate ratio.

**Table 2:** Differences in MRI-associated data between the anaesthesia/sedation and FWT group

	Sedation (*n* = 243)	Feed-and-wrap (*n* = 189)	Total (*n* = 432)	*p* value
**Length of the examination (min)**				
mean ± sd	39 ± 14	32 ± 12	36 ± 14	**< 0.001**
**Field strength**				
1.5 T	95 (39%)	65 (34%)	160 (37%)	0.366
3 T	148 (61%)	124 (66%)	272 (63%)	
**Body region**				
Head	138 (57%)	122 (65%)	260 (60%)	0.096
Neck/thorax	5 (2%)	6 (3%)	11 (2%)	
Abdomen	30 (12%)	28 (15%)	58 (13%)	
Musculoskeletal	19 (8%)	8 (4%)	27 (6%)	
Whole-body/multiple regions*	51 (21%)	25 (14%)	76 (18%)	
**Indication**				
Neurological	132 (54%)	117 (62%)	249 (58%)	0.086
Oncological	42 (17%)	19 (10%)	61 (14%)	
Visceral	15 (6%)	19 (10%)	34 (8%)	
Cardiovascular	1	2 (1%)	3 (1%)	
Other	17 (7%)	12 (6%)	29 (7%)	
Multiple	36 (15%)	20 (11%)	56 (13%)	
**Administration of contrast agent**				
No	169 (70%)	177 (94%)	346 (82%)	**< 0.001**
Yes	74 (30%)	12 (6%)	86 (20%)	
**Total number of sequences (t)**				
Mean ± SD	8.9 ± 3	8.1 ± 2.4	8.5 ± 2.8	**0.023**
**Number of repeated sequences**				
Mean ± SD	0.13 ± 0.43	0.24 ± 0.56	0.18 ± 0.49	**0.007**
**Reason for sequence repetition**				
No Sequence repeated	220 (91%)	154 (81%)	374 (87%)	**0.015**
Technical	3 (1%)	6 (3%)	9 (2%)	
Patient motion	17 (7%)	28 (15%)	45 (10%)	
Other	3 (1%)	1 (1%)	4 (1%)	
Number of sequences with diagnostic limitations** (N)				
Mean ± SD	0.07 ± 0.31	0.49 ± 0.96	0.25 ± 0.59	**< 0.001**
**Incidence rate (= N/t)**				
Mean ± SD	0.0077 ± 0.0033	0.07 ± 0.16	0.035 ± 0.07	**< 0.001**
**Sequence quality score**				
Mean ± SD	3.96 ± 0.11	3.7 ± 0.43	3.9 ± 0.32	**< 0.001**

Significant differences are marked in bold. *whole-body (*n* = 35), head and spine (*n* = 17), head and neck/thorax (*n* = 6), abdomen and spine (*n* = 6), head and abdomen (*n* = 4), neck/thorax/abdomen (*n* = 4), head and musculoskeletal (*n* = 3), abdomen and musculoskeletal (*n* = 12), ** defined as quality score lower than 3. sd = standard deviation

**Table 3:** Estimated incidence rate ratios derived from negative binomial model

Predictor	Incidence rate ratio (95%CI)	*p* value
**Technique**		
FWT versus sedation	8.74 (6.73–11.37)	**< 0.001**
**Corrected age [days]**	1.006 (1–1.012)	**0.048**
**Gender**		
Male versus female	0.95 (0.76–1.29)	0.948
**Body weight [kg]**	0.86 (0.73–1.02)	0.086
**Body region**		
Neck/thorax versus head	1.91 (0.89–4.07)	0.096
Abdomen versus head	0.46 (0.31–0.68)	**< 0.001**
Musculoskeletal versus head	0.63 (0.35–1.15)	0.132
Whole-body/multiple regions versus head	0.33 (0.22–0.48)	**< 0.001**
**Field strength**		
3 T versus 1.5 T	1.23 (0.94–1.6)	0.129

### Subgroup analysis of patients examined by the FWT

**Fig. 4:**
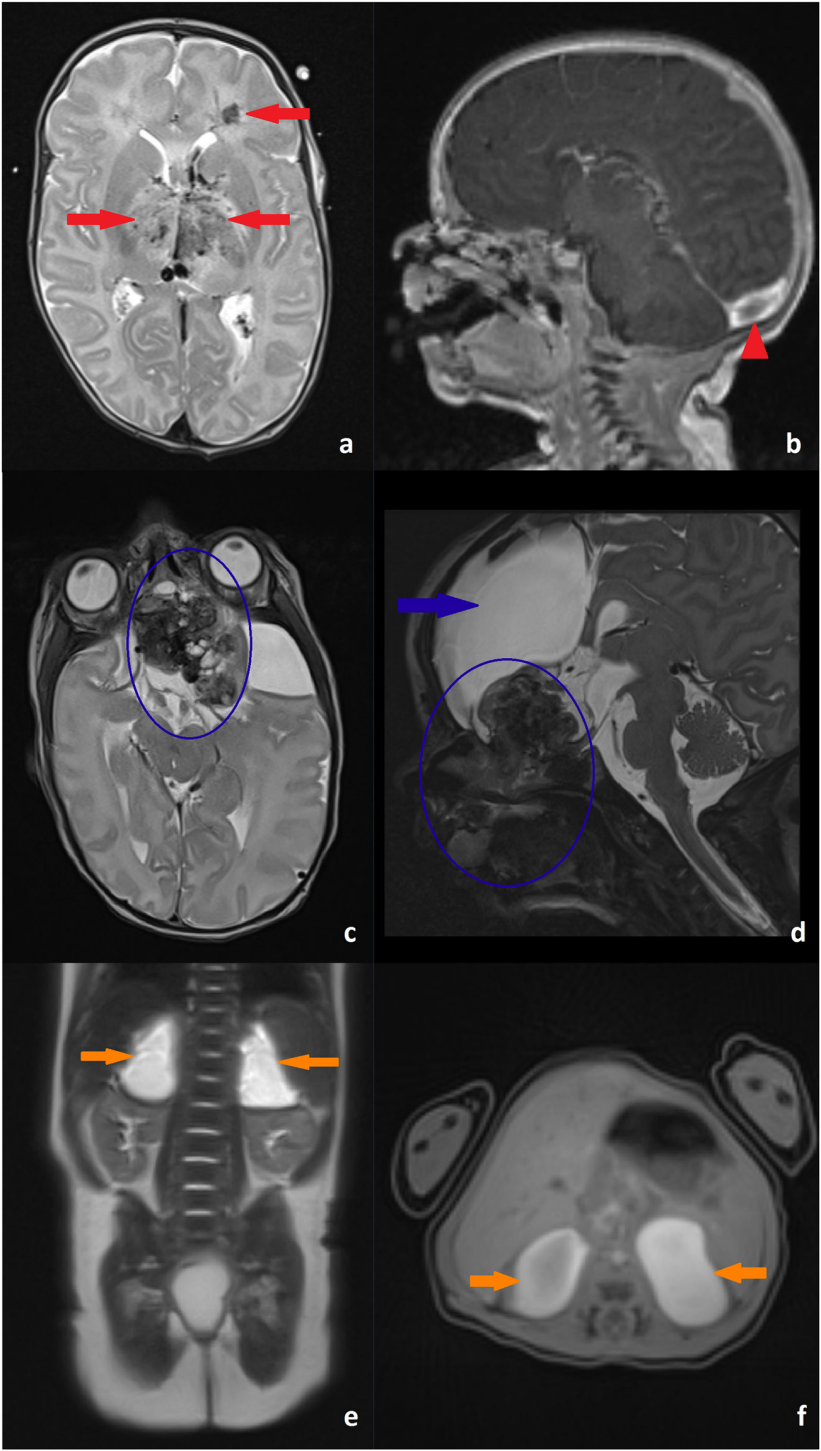
Clinical examples of examinations performed using the FWT. **a** T2-weighted turbo spin echo sequence demonstrating venous congestion bleeding in the periventricular white matter and basal ganglia (red arrows) due to (**b**) thrombosis in the sinus confluens (red arrowhead, T1-MPRAGE (Magnetization Prepared Rapid Acquisition with Gradient Echoes) postcontrast) in a 13-day old female patient. T2-weighted turbo spin echo (**c**) and 3D-SPACE (Sampling Perfection with Application optimized Contrast using different flip angle Evolution) (**d**) sequence demonstrating a large craniofacial teratoma (blue circles) with a large adjacent intracranial arachnoid cyst (blue arrow) in a 4-day-old female patient. T2-weighted Half fourier Single-shot Turbo spin-Echo (**e**) and T1-weighted 3D stack of stars gradient echo sequence (**f**) confirming suspected bilateral adrenal gland bleeding (orange arrows) in a 7-day old male patient

**Fig. 5:**
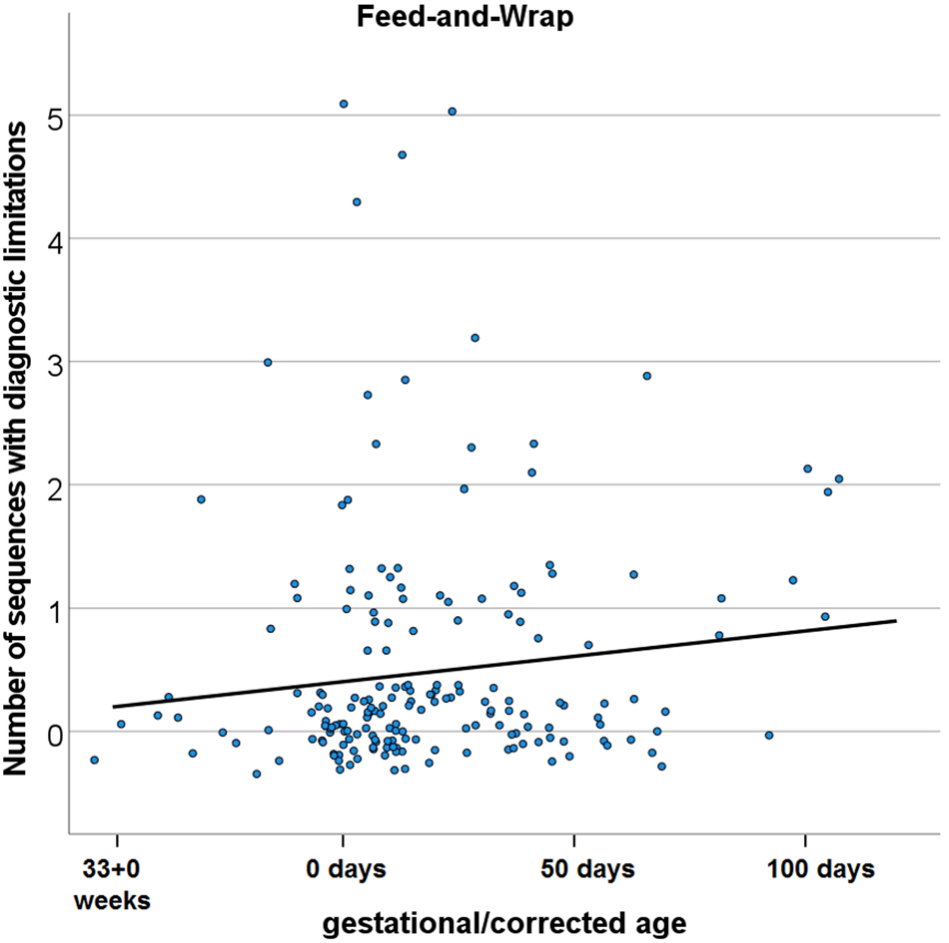
Number of sequences with diagnostic limitations depending on corrected age in the “feed-and-wrap” group. We applied a jitter technique to avoid multiple overlapping data points in order to enhance the visibility and interpretability of the data

Clinical examples of successful examinations in the FWT group are demonstrated in Fig. 4. Mean sequence quality did not differ significantly between term and preterm patients (mean ± SD: 3.8 ± 0.48 versus 3.7 ± 0.42, *p* = 0.108). However, image quality decreased significantly with increasing corrected age (rho = -0.18, *p* = 0.013). In addition, multivariate negative binomial regression analysis demonstrated an increase in the number of sequences with diagnostic limitations with increasing age in the FWT group (Fig. 5).

## Discussion

The results of this study demonstrated, in a large patient collective, that the FWT is a feasible and safe method to perform MRI examinations in neonates, even in low-weight preterm patients. The drawbacks include a slightly lower image quality and a higher probability of sequence repetitions. However, this did not lead to longer examination times. In contrast, total examination times were significantly shorter when using the FWT, most probably due to more focused MR protocols, while the clinical question could still be answered in the majority of cases (95%). Optimal sequence quality was achieved in 82.6% of all sequences acquired in the FWT group (and 97.4% under sedation).

Several authors have assessed the success rates of the FWT or equivalent techniques. Success rates are defined by achieving diagnostic image quality in a single FWT attempt. However, these techniques are inconsistently used, as demonstrated in a recent survey study [20]. One early report stated an 89% success rate in a cohort of 39 patients [21]. More recently, Templeton et al [11] reported success rates between 91.1 and 95% (depending on the age of the child) for neonatal brain MRI in a cohort of 217 patients. Antonov et al [14] evaluated a cohort of 279 patients, including examinations of different body regions. The clinical question could be completely addressed in 79% of the cases in this study. Caro-Dominguez et al [22] assessed success rates and sequence quality in a small study of 47 brain MR examinations using a feed-and-sleep technique versus 47 controls (using sedation). The success rate for the “feed-and-sleep” group was 89%, and “optimal” quality was achieved in 60% of the cases (versus 89% in the sedation group). Two groups have assessed cardio-vascular “feed-and-sleep” MRI, first, in a cohort of 20 [15] and second, in a cohort of 60 patients [4], both reporting a 100% success rate. The technique could, therefore, potentially be adapted for novel advanced MR methods, such as cardiac 4D flow MRI [23]. Tsiflikas et al [16] were able to demonstrate 90% successful functional contrast-enhanced MR urographies in a cohort of 42 children younger than 1 year. They also compared image quality to a control group using sedation, which contained 19 patients. Sequence quality was significantly higher in the latter group. However, all completed examinations in the “feed-and-sleep” group were diagnostic.

In terms of the overall success rate of the FWT, which was 95% in this study, our results are in line with most of the aforementioned publications. In the literature, the rate ranged between 89 and 100%. Only one, though relatively large, study [14] presented a lower amount of completely conclusive examinations (79%). However, an additional 20% of examinations at least partially addressed the clinical question. Uncertainty remains if a failed FWT attempt should be repeated using the same technique. Data from the aforementioned survey study [20] suggests that most centres (35%) switch to sedation, but a minority (4%) reported up to three FWT attempts. However, there is a lack of evidence concerning the optimal number of non-sedated MRI attempts before switching to sedation.

When “feed-and-wrap” techniques were introduced, many studies concentrated on brain MRI. In our study, brain MRI also accounted for the majority of examinations (64% in the FWT group). Hence, in approximately one-third of the cases, other body parts were imaged, including whole-body MRI. Thus, an important aspect of this study, in line with others [24], is that not only the brain but also other regions of interest can be reliably scanned with comparable rates of success. Our multivariate regression model even demonstrated a lower portion of sequences with diagnostic limitations when imaging other body parts (abdomen, whole-body MRI) compared with the head (Table 3). This result might partly be explained by the fact that in body imaging, other artefacts, e.g., technically unpreventable breathing artefacts, lower the overall impression more than motion artefacts. Therefore, the judging radiologist might have determined a higher quality score as some artefacts are expected and tolerated. In addition, single-shot sequences (e.g., for abdominal imaging) are not as prone to motion artefacts compared with sequences used for brain imaging.

While many of the studies primarily assessed success rates of the whole MR examination, image quality evaluated per sequence was rarely reported. Our study demonstrated a slightly lower mean sequence quality when using the FWT compared with sedation. Adapted to our scale, a mean score of 3.6 for the FWT group and 3.9 for the sedation group can be derived from the presented data of the study of Caro-Dominguez et al [22]. These data are nearly equivalent to the results of our study (3.7 versus 3.96). Tsiflikas et al [16] presented similar results (derived mean score: 2.5 versus 3 on a 3-point scale), which are difficult to quantitatively compare with our study because of the different scale.

As sedation risks increase in immature preterm patients, especially with low body weight, the FWT is a reliable option to perform MRI studies in this age group. This particularly applies to patients who do not require mechanical ventilation at the intensive care unit. In our cohort, the lowest gestational age at the time of the MRI scan in the FWT group was 33 + 1 weeks, and the lowest body weight was 1.6 kg. Thus, a special focus on preterm patients is of interest. Our results are partly in line with the study of Templeton et al [11], who describe a nearly equal success rate in preterm and term patients below 90 days of age (92.5 versus 91.1%). Correspondingly, in our study, mean sequence quality did not differ significantly between term and preterm patients. Interestingly, Templeton et al describe a higher success rate (95%) in older term patients (90–181 days of age). This does not correspond to our clinical experience and data, as sequence quality slightly decreased with the increasing age of the child in the FWT group, and an increase in incidence rates with increasing corrected age was demonstrated in the multivariate analysis.

## Limitations

The limitations of our study include the retrospective study design and consecutive long covered time span from 2009 until 2022. This inherently caused changes of the MR scanner and/or protocols. In particular, the 3 T scanner was replaced in 2019. As some faster sequences were available, this could have led to shorter examination times. The 1.5 T scanner was continuously used throughout the study period without major protocol changes. Lower rates of contrast agent application in the FWT group are partly explained by the general approach to avoid gadolinium-based agents whenever possible (due to reasons like nephropathy or gadolinium deposition in the central nervous system). In addition, the individual reasons for the decision of whether to perform the examinations under sedation or using the FWT were not documented in the medical records. Thus, we cannot exclude that the responsible radiologist might have favoured a sedation setup when there was a high probability of contrast media necessity.

## Conclusions

In conclusion, the results of this study demonstrated in a large patient cohort that the FWT is a feasible and safe method for neonatal MRI, especially in low-weight premature patients. Imaging can be performed with a high success rate and only slightly lower image quality compared with examinations under sedation. This is of high clinical relevance, as deep sedation may be of high risk in the preterm and neonatal age group due to its short- and potential long-term complications. Thus, especially in immature, low-weight preterm patients, the FWT is a reliable option to perform MRI studies without exposing the child to risks associated with sedation.

## Supplementary information

Electronic Supplementary Material

## Abbreviations

FWT: “Feed-and-wrap” technique
MR(I): Magnetic resonance (imaging)
SD: Standard deviation
