# Supplementary material for: “Feed-and-wrap” technique versus deep sedation for neonatal magnetic resonance imaging: a retrospective comparative study
Source: Eur Radiol. 2024 May 7;34(11):7104–14. doi: 10.1007/s00330-024-10777-6 (PMC11519104; doi:10.1007/s00330-024-10777-6)
Supplement: Supplementary file 1 — Electronic Supplementary Material [file 330_2024_10777_MOESM1_ESM.pdf]

“Feed-and-wrap” technique versus deep sedation for neonatal magnetic resonance imaging: a retrospective comparative study

ELECTRONIC SUPPLEMENTARY MATERIAL

Supplementary Table 1 – Differences in total examination length, analysis of different subgroups based on the use of contrast agent and body region.

**Length of the examination (min)**	**Sedation**	**„Feed-and-Wrap“**	**Total**	***p*- value**
All patients	39 ± 14 (n = 243)	32 ± 12 (n = 189)	36 ± 14 (n = 432)	**< 0.001**
Without contrast agent	36 ± 13 (n = 169)	31 ± 11 (n = 177)	34 ± 13 (n = 346)	**< 0.001**
With contrast agent	44 ± 15 (n = 74)	42 ± 17 (n = 12)	44 ± 15 (n = 86)	0.995
Head	38 ± 10 (n = 138)	32 ± 9 (n = 122)	35 ± 10 (n = 260)	**< 0.001**
Neck/Thorax	37 ± 7 (n = 5)	33 ± 21 (n = 6)	34 ± 15 (n = 11)	0.429
Abdomen	31 ± 12 (n = 30)	22 ± 9 (n = 28)	26 ± 11 (n = 58)	**0.001**
Musculoskeletal	28 ± 12 (n = 19)	30 ± 16 (n = 8)	28 ± 13 (n = 27)	0.897
Whole-body/multiple regions	50 ± 19 (n = 51)	40 ± 15 (n = 25)	47 ± 18 (n = 76)	**0.043**

All values are presented as mean ± standard deviation, and total numbers of patients in the respective subgroup (in brackets). Significant differences are marked in bold.

Supplementary Table 2 – Estimated incidence rate ratios derived from negative binomial model, subgroup analysis based on corrected age.

**Predictor**	**Incidence rate ratio (95%-confidence interval)**	***p*- value**
**Technique**<br>FWT versus sedation	 3.39 (1.47 – 7.83)	 < **0.001**
**Corrected age [days]**	1.014 (1.005 – 1.024)	**0.004**
**Gender**<br>male versus female	 0.96 (0.72 – 1.3)	 0.964
**Body weight [kg]**	0.7 (0.54 – 0.9)	**0.006**
**Body region**<br>neck/thorax versus head<br>abdomen versus head<br>musculoskeletal versus head<br>whole-body/multiple regions versus head	 0.52 (0.22 – 1.25)<br>0.56 (0.35 – 0.89)<br>0.86 (0.43 – 1.74)<br>0.44 (0.29 – 0.69)	 0.521<br>**0.015**<br>0.682<br>< **0.001**
**Field strength**<br>3T versus 1.5T	 1.12 (0.83 – 1.51)	 0.442

a) Results of subgroup 1 (corrected age of 50 days or lower, n = 292)

**Predictor**	**Incidence rate ratio (95%-confidence interval)**	***p*- value**
**Technique**<br>FWT versus sedation	 162 (63 – 420)	 < **0.001**
**Corrected age [days]**	1.072 (1.042 – 1.1)	< **0.001**
**Gender**<br>male versus female	 1.57 (0.58 – 4.22)	 0.342
**Body weight [kg]**	0.57 (0.36 – 0.9)	**0.016**
**Body region**<br>neck/thorax versus head<br>abdomen versus head<br>musculoskeletal versus head<br>whole-body/multiple regions versus head	 n/a (low sample size)<br>0.04 (0.01 – 0.19)<br>n/a (low sample size)<br>0.04 (0.01 – 0.15)	 <br>< **0.001**<br><br>< **0.001**
**Field strength**<br>3T versus 1.5T	 0.27 (0.11 – 0.68)	 **0.005**

b) Results of subgroup 2 (corrected age > 50 days, n = 126, 14 cases – neck/thorax and musculoskeletal – excluded due to low sample size for body region)
